# The PAL test for malignant disease in man.

**DOI:** 10.1038/bjc.1978.42

**Published:** 1978-02

**Authors:** W. A. Schottler, H. W. Bauer


*Br. J. Cancer* (1978) **37**, 319

***Short Communication***

### THE PAL TEST FOR MALIGNANT DISEASE IN MAN

W. AX, S. SCHOTTLER AND H. W. BAUER

*From the Behringwerke AG, 355 Marburg, Germany*

Received 3 October 1977 Accepted 24 October 1977

It was recently shown that the improvement of the histone-induced agglutination of lymphocytes (Sabolovic *et al.*, 1975) by synthetic peptides (Bauer and Ax, 1977) might also be of interest in detecting malignancies. As opposed to all other tests mentioned, the poly-L-lysine-induced agglutination test (PAL test) is simple, rapid and requires a minimum of laboratory practice. By screening a large population the present study should confirm the extent of agreement between microagglutination induced by poly-L-lysine of mol. wt ~3400 and malignant growth.

*Patients*.—Studies were performed on 300 persons of both sexes and of all ages with the PAL test described in detail earlier (Bauer and Ax, 1977). Ninety of them had malignant tumours, 140 had non-malignant or pre-malignant diseases. For the purpose of further comparison, 70 healthy controls were also tested. All 3 groups were of comparable range of age and sex. Patients with non-malignant diseases were mainly those with acute or chronic conditions, especially inflammations of the gastrointestinal and urogenital tracts, the lung and the bile-duct system.

*Lymphocytes*.—Lymphocytes were prepared from heparinized or citrated blood by Ficoll-Isopaque® gradient as described by English and Anderson (1974) using 2 layers of separating fluid of sp.gr. (a) 1·077 and (b) 1·119 at 25°C. The plasma–solution (a) interface consists of lymphocytes (90%), solution (a)–(b) interface of polymorphonuclear cells (80%).

*Test procedure*.—1 ml of lymphocytes, diluted with Hanks' solution to a concentration of $6 \times 10^6$/ml, was mixed with 1 ml of basic peptide poly-L-lysine mol.wt 3400 of 0·05 mg/ml dissolved in 0·145M NaCl, pH 7·0. The mixture was distributed into microplates, 20 μl per well, and incubated for 30 min at 37°C in a humid atmosphere.

*Results*.—Of the 70 normal subjects, none showed positive results in the test (Table I). In the 140 patients with non-malignant disease the blood lymphocytes showed positive reactions in 24% = 33/140 cases (Table I). By contrast, 81% = 73/90 cases of the patients with neoplastic disease showed agglutination (Table II). Negative reactions were found mainly in patients with advanced metastases. We did not find a difference between patients with a tumour and those who were tumour-free long after surgery (6 cases, 1–12 months after tumour resection).

When the reaction with the poly-L-lysine fraction was very strong, all cells were agglutinated, but if the cell concentration was reduced below the value given in the test procedure above, no agglutination occurred at all. This demonstrates the importance of a sufficient lymphocyte concentration per well for cells to contact each other, before a positive reaction can occur. The extended findings of the test described here seem to confirm the value of this procedure for detecting "malignancy" in man. A relatively simple "blood-test" for neoplasm is of considerable interest as a scanning procedure.

TABLE I.—*Poly-L-lysine Agglutination (PAL) Test in Patients with Non-malignant Diseases and Healthy Controls*

| Peripheral lymphocytes from patients with | Results + | − | Total |
|---|---|---|---|
| Cholecystopathy | 8 | 11 | 19 |
| Diabetes mellitus | 0 | 13 | 13 |
| Appendicitis | 2 | 9 | 11 |
| Ulcerative colitis | 4 | 2 | 6 |
| Hernia inguinalis | 2 | 5 | 7 |
| Goitre and hyperthyroidism | 1 | 6 | 7 |
| Pulmonary embolus | 0 | 4 | 4 |
| Pneumonia + chronic bronchitis | 1 | 4 | 5 |
| Nephritis | 3 | 6 | 9 |
| Rectal polyp | 3 | 2 | 5 |
| Hypertonus | 1 | 5 | 6 |
| Rheumatoid arthritis | 1 | 6 | 7 |
| Attempted suicide (barbiturate) | 0 | 1 | 1 |
| Generalized arteriosclerosis + heart attack | 0 | 10 | 10 |
| Haemorrhoids | 1 | 3 | 4 |
| Mononucleosis | 0 | 2 | 2 |
| Prostatic adenoma | 0 | 1 | 1 |
| Porphyria cut. tarda | 0 | 1 | 1 |
| Pancreatitis, chronic | 3 | 0 | 3 |
| Diverticulitis | 0 | 2 | 2 |
| Eczema | 1 | 1 | 2 |
| Lymphoma, benign | 1 | 1 | 2 |
| Thalassaemia | 1 | 0 | 1 |
| Ovarian cyst | 0 | 1 | 1 |
| Hepatitis | 0 | 2 | 2 |
| Ulcus ventriculi | 0 | 1 | 1 |
| Hypogonadism | 0 | 1 | 1 |
| Morbus Werlhof | 0 | 1 | 1 |
| Stomatitis | 0 | 1 | 1 |
| Polyneuropathy | 0 | 1 | 1 |
| IgA-Plasmocytoma | 0 | 1 | 1 |
| Mucocoele | 0 | 1 | 1 |
| Abscess | 0 | 1 | 1 |
| Trauma | 0 | 1 | 1 |
| Total | 33 (24%) | 107 (76%) | 140 |
| HEALTHY CONTROLS | 0 | 70 | 70 |

TABLE II.—*Poly-L-lysine Agglutination (PAL) Test in Cancer Patients*

| Site of carcinoma | Results + | − | Total |
|---|---|---|---|
| Thyroid | 2 | 1 | 3 |
| Bronchus | 4 | 2 | 6 |
| Breast | 8 | 3 | 11 |
| Stomach | 10 | 2 | 12 |
| Pancreas | 4 | 1 | 5 |
| Colon, sigmoid | 12 | 2 | 14 |
| Rectum | 14 | 1 | 15 |
| Kidney | 5 | 1 | 6 |
| Prostate | 1 | 0 | 1 |
| Corpus uteri | 2 | 0 | 2 |
| Ovary | 2 | 0 | 2 |
| Testis | 1 | 0 | 1 |
| Malignant lymphoma | 5 | 1 | 6 |
| Melanoma | 2 | 2 | 4 |
| Sarcoma | 1 | 1 | 2 |
| Total | 73 (81%) | 17 (19%) | 90 |

Despite unexpected findings, we are confident of our results, even though we can still present no satisfactory explanation for the underlying mechanism(s).

It is a pleasure to acknowledge the helpful cooperation of the surgeons (Professor H. Richter and Professor G. Rodeck) and the physicans (Dr W. D. Gassl and Dr G. Walch) of the Universitätsklinikum Marburg for blood samples and access to patients.

### REFERENCES

BAUER, H. W. & AX, W. (1977) Detection of Sensitized Human Blood Lymphocytes by Agglutination with Basic Peptides: a Possible Test for Malignant Disease. *Br. J. Cancer*, **36**, 708.

ENGLISH, D. & ANDERSON, B. R. (1974) Single-step Separation of Red Blood Cells, Granulocytes and Mononuclear Leukocytes on Discontinuous Density Gradients of Ficoll Hypaque. *J. immunol. Meth.*, **5**, 249.

SABOLOVIC, D., SABOLOVIC, N., MOUTTE, A., LEIBOVICI, S., SAUVEZIE, B., CHOLLET, P. & PLAQUE, R. (1975) Agglutination of Peripheral Blood Lymphocytes from Cancer Patients and not from Healthy Controls with the $F_{2a1}$ Histone Fraction. *Br. J. Cancer*, **32**, 28.


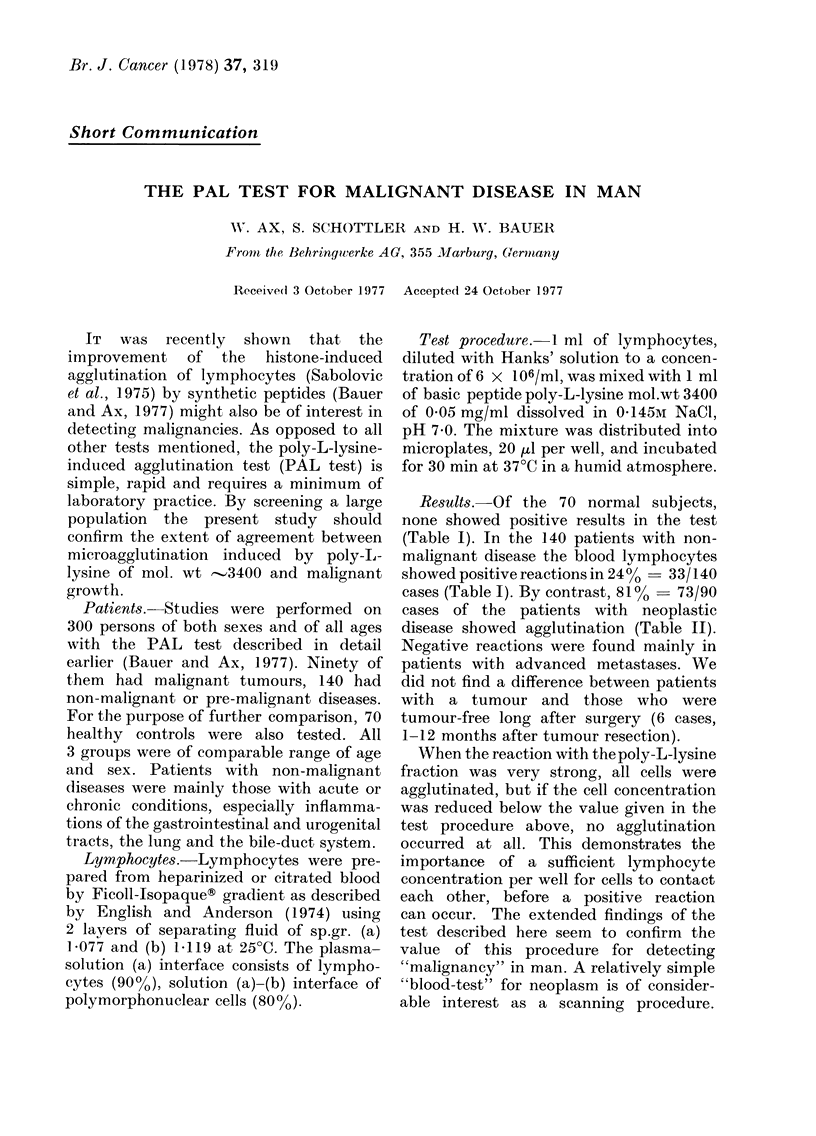

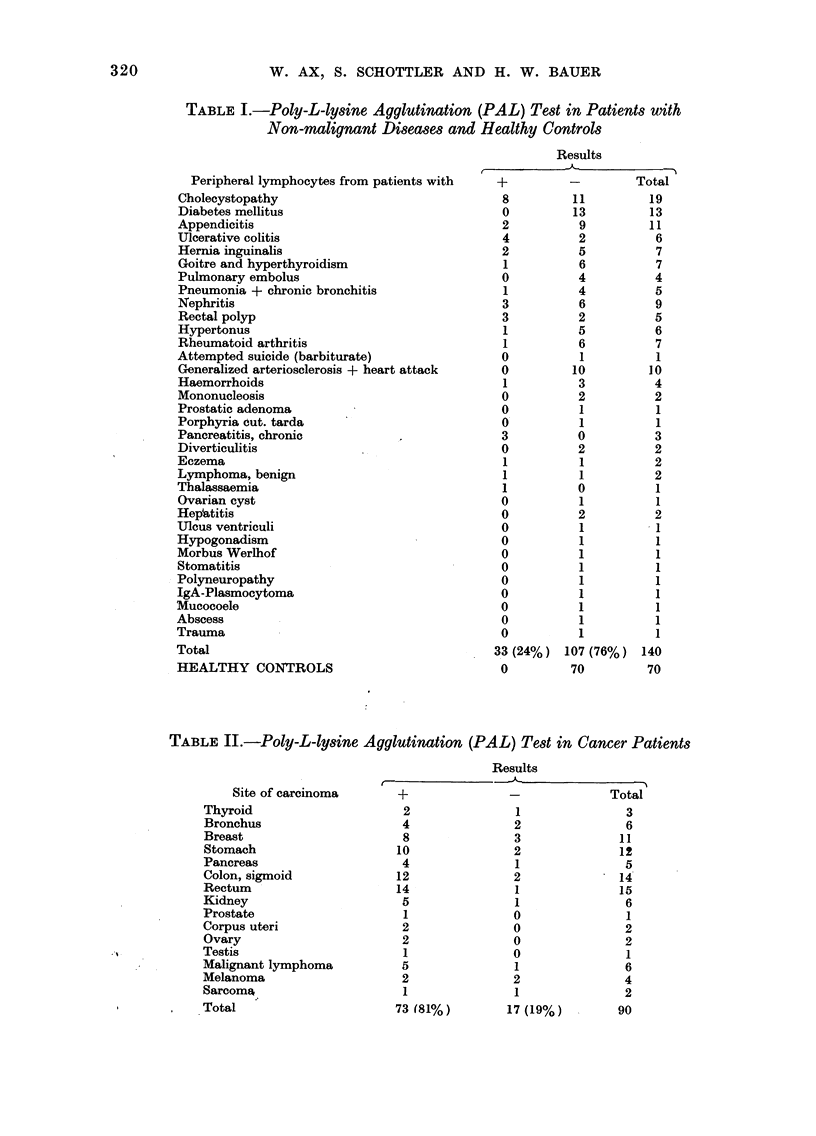

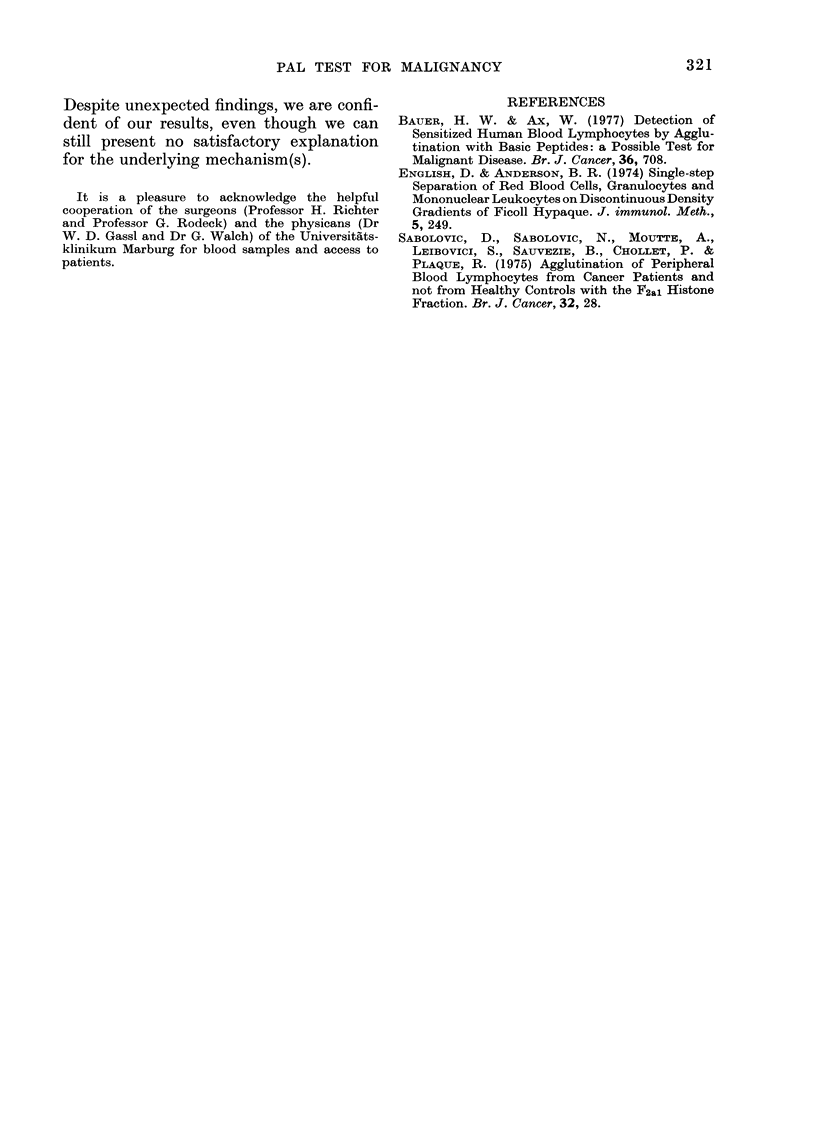


## References

[OCR_00334] Bauer H. W., Ax W. (1977). Detection of sensitized human blood lymphocytes by agglutination with basic peptides: a possible test for malignant disease.. Br J Cancer.

[OCR_00340] English D., Andersen B. R. (1974). Single-step separation of red blood cells. Granulocytes and mononuclear leukocytes on discontinuous density gradients of Ficoll-Hypaque.. J Immunol Methods.

[OCR_00347] Sabolović D., Sabolović N., Moutte A., Leibovici S., Sauvezie B., Chollet P., Plagne R. (1975). Agglutination of peripheral blood lymphocytes from cancer patients and not from healthy controls, with the F2A1 histone fraction.. Br J Cancer.

